# MRI scan with the “feed and wrap” technique and with an optimized anesthesia protocol: a retrospective analysis of a single-center experience

**DOI:** 10.3389/fped.2024.1415603

**Published:** 2024-08-23

**Authors:** Giulia Moltoni, Giulia Lucignani, Stefania Sgrò, Alessia Guarnera, Maria Camilla Rossi Espagnet, Francesco Dellepiane, Chiara Carducci, Stefano Liberi, Elisabetta Iacoella, Giuliana Evangelisti, Anna Contini, Francesca Campi, Immacolata Savarese, Carlo Gandolfo, Daniela Longo

**Affiliations:** ^1^ Functional and Interventional Neuroradiology Unit, Bambino Gesù Children’s Hospital, IRCCS, Rome, Italy; ^2^ Department of Neuroradiology, NESMOS S.Andrea Hospital, University Sapienza, Rome, Italy; ^3^ Department of Anesthesia and Critical Care, Bambino Gesù Children’s Hospital, IRCCS, Rome, Italy; ^4^ Department of Imaging, Bambino Gesù Children’s Hospital, IRCCS, Rome, Italy; ^5^ Nuclear Medicine and Advanced Oncological Imaging Unit, Bambino Gesù Children’s Hospital, IRCCS, Rome, Italy; ^6^ Neonatal Intensive Care Unit, Bambino Gesù Children’s Hospital—IRCCS, Rome, Italy

**Keywords:** MRI, newborn, children, feed and wrap, anesthesia

## Abstract

**Introduction:**

MRI examinations in the pediatric population require acquiring motionless images in the safest possible manner. At our institute, we have developed a protocol called “Good Practice” aimed at avoiding anesthesia in newborns and infants through the use of the “feed and wrap” technique, as well as preventing hospitalization for older children requiring anesthesia with an optimized sedation protocol. We evaluated this protocol in terms of patient safety, imaging quality, and parental satisfaction.

**Materials and methods:**

Patient data were collected retrospectively. In the feed and wrap group, image quality and the necessity of repeating the examination were evaluated. In the optimized anesthesiologic protocol group, various parameters were analyzed to assess the safety of the protocol. Parental satisfaction was determined through a questionnaire.

**Results:**

A total of 132 patients were included, with 82 undergoing the feed and wrap technique and 50 receiving the optimized anesthesiologic protocol. In the feed and wrap group, images were classified as follows: 4.87% poor, 18.29% sufficient, 37.80% good, and 39.92% excellent. In only 2 cases a new MRI examination was required. In the optimized anesthesiologic protocol group, no adverse effects were observed, and no post-anesthesia hospitalizations were needed. 100% of parents of babies examined with the feed and wrap technique rated it as excellent. Furthermore, 85.6% of parents considered the optimized anesthesiologic protocol excellent, and 13.6% rated it as good.

**Conclusion:**

At our institute, the feed and wrap technique proved to be effective in obtaining high-quality images. Anesthesia using propofol showed no adverse effects and proved to be successful in avoiding hospitalization. Parents expressed relief at the avoidance of anesthesia and hospitalization for their children.

## 1 Introduction

Performing MRI examinations in the pediatric population presents unique challenges compared to adults, as obtaining motionless images can be quite difficult. This often necessitates procedural sedation, which can increase parental anxiety, hospitalization rates, and healthcare costs.

The safety of general anesthesia in children under three years old has been a topic of debate in recent literature. While many studies suggest that early exposure to anesthesia may not negatively impact neurocognitive outcomes, others suggest that an impact may be present and there is ongoing interest in finding ways to avoid or optimize anesthesia protocols for neonates and young children in clinical practice (1–4).

One technique that has gained attention is the “feed and wrap” method, which allows for MRI scanning in newborns without need of anesthesia. With this approach, parents feed the neonate in a comfortable setting before the scan to induce natural sleep, followed by swaddling to reduce motion artifacts. This technique eliminates the need for anesthesia and allows neonates to return to their parents immediately after the scan. However, challenges include the risk of awakening the newborn during the scan, potential motion artifacts, and the need for a skilled nursing team to monitor vital signs and prevent overheating or excessive swaddling.

For infants older than three months or newborns weighing over 3–4 kg, the feed and wrap technique may not be feasible, and anesthesia with sevoflurane inhalation is often used. However, sevoflurane anesthesia can lead to post-anesthetic symptoms such as agitation, nausea, and vomiting, which necessitate day-hospital surveillance (5).

An alternative is the use of propofol, an intravenous anesthetic with fast onset, rapid recovery, and minimal side effects like nausea and vomiting (6).

We conducted a retrospective evaluation of the “Good Practice” protocol used in our Institute, which aims to avoid general anesthesia in newborns and infants using the feed and wrap technique, and to optimize anesthesia with propofol in older infants and children.

Our study aimed to assess the effectiveness of the protocol used in our Institute in terms of MRI image quality, anesthesia-related side effects, and parental satisfaction, aiming to provide safe and comfortable MRI examinations for these young patients while reducing hospitalization and parental distress.

## 2 Material and methods

Patients who underwent the “Good Practice” protocol at our institute between March 2020 and December 2022 were retrospectively recruited through a review of the image archive and clinical records.

The “Good Practice” protocol is a standardized anesthesiologic and radiological protocol at our institute. It involves acquiring MRI examinations at 3T Siemens Magnetom Vida or Skyra scanners, using natural sleep with the feed and wrap technique for newborns and infants under 3 months of age and weighing less than 4 kg. In patients older than 3 months or weighting more than 4 kg, the feed and wrap technique may result not effectiveness because they tend not to maintain a prolonged sleep after feeding or may not be comfortable in the vacuum bag because of their size. Therefore, in young children, or newborns and infants who do not meet the criteria for the feed and wrap method due to age, weight or contraindications (see below), an anesthesiologic procedure sedation with propofol is performed without the need for hospitalization.

The feed and wrap technique, as its name suggests, comprises two main phases. The first one is the “feed”: just before the scan, the baby is fed by either their parent or a nurse in a room adjacent to the MRI suite (called the recovery room). A pacifier may be used if necessary to help the baby fall asleep naturally, and it can remain in the baby's mouth during the scan to maintain sleep. Babies also wear earplugs secured with tape to minimize external noise.

The second phase is the “wrapping”: the patient is carefully swaddled first in a blanket (inner layer) and then in a vacuum bean bag (outer layer). At our institute, we use a MedVac® device for this purpose. After wrapping the vacuum bag around the infant, the straps are secured, and the bag is connected to a pump. The air is then removed from the bag just before transporting the baby into the scanner room. This process allows the bag to conform snugly around the newborn without exerting pressure. If necessary, the baby may be fed again before the scanning procedure commences.

The heart rate, oxygen saturation and capnography are continuously monitored throughout the scan. Pulse oximetry is attached to the patient's foot before completing the swaddling process. If contrast injection is required, the contrast syringe is connected to the infant's intravenous access before the scan begins; the intravenous line is usually previously insert before the feeding phase. The temperature in the MRI gantry is carefully regulated using a ventilation system to prevent overheating of the swaddled baby.

Contraindications for the feed and wrap procedure include feeding difficulties, risk of respiratory compromise, or patients who are intubated. Therefore, before the procedure newborns must be carefully evaluated by a neonatologist and an anesthetist.

The optimized anesthesiologic protocol used in our institute is based on emerging literature regarding the safety of propofol as an anesthetic in children (5, 7, 8). The previous protocol was based on inhalator anethetics, such as sevoflurane, and it did not include a premedication. The optimized anesthesiologic protocol used in our institute includes:

1. Premedication with midazolam at a dose of 0.4 mg/kg orally administered 40 min before the MRI examination.
2. Application of EMLA© cream on children hands to facilitate the insertion of an intravenous line before anesthesia induction.
3. Intravenous anesthesia induction with a bolus injection of 1.5–2.5 mg/kg of propofol, slowly administered, based on patient's age (refer to Table 1).
4. Maintenance of anesthesia with a propofol infusion at a rate of 5–8 mg/kg/h. The propofol dose is modulated according to blood pressure and heart rate.

**Table 1:** Optimized anesthesiologic protocol based on age.

Age	Premedication (Midazolam oral)	Induction (Propofol iv)	Maintaining (Propofol iv)
Newborns (0–3 months)		2.5 mg/kg	5–8 mg/kg/h
Infants (3 months–1 year)	0.4 mg/kg	2 mg/kg	5–8 mg/kg/h
Children (>1 year)	0.4 mg/kg	1.5 mg/kg	5–8 mg/kg/h

During MRI examination, respiratory support is provided using oxygen and air assistance by face mask, while the patient maintains spontaneous breathing. Continuous monitoring of heart rate, oxygen saturation respiratory rate, EtCO2, and graphical waveform is conducted throughout the scan. Anesthesia lasts for the entire duration of the MRI examination, with an average of about 35–40 min.

Contraindications for using propofol include known hypersensitivity to propofol or any components of the propofol injectable emulsion (such as eggs, egg products, soybeans, or soy products), heart diseases, and excessively low blood pressure (9, 10). In these cases, patients are anesthetized with the commonly used protocol based on Sevoflurane.

### 2.1 Data collection and analysis

#### 2.1.1 Feed and wrap technique

Medical data for each subject were collected from clinical records, radiology reports, and the imaging archive. This included patient's characteristics such as gender, age in days at the time of scan, gestational age, clinical indication for MRI, and post-MRI complications. Additionally, the body part scanned, and the use of contrast were documented (refer to Table 2).

**Table 2:** Feed and wrap group characteristics.

	Descriptive Statistics
Gender	
- Male	48 (58.54%)
- Female	34 (41.46%)
Age (days)	
- Media	50
- Range	1–120
- Standard deviation	37.8
Preterm	8 (9.75%)
Body part	
- Brain	70 (85.36%)
- Spine	6 (7.32%)
- Head and neck	6 (7.32%)
Contrast-medium administration	7 (8.5%)
Indication for scan	
- Hypoxic-ischemic brain insult	28 (34.15%)
- Congenital or neonatal infection	12 (14.63%)
- Metabolic disorders	10 (12.19%)
- Neurological symptoms	20 (24.39%)
- Spinal dysraphism	6 (7.32%)
- Head&neck district pathologies	6 (7.32%)

For patients who underwent the feed and wrap technique, two pediatric neuroradiologists (G.M. with 5 years of experience and D.L. with 30 years of experience) reviewed the MRI examinations. They provided a consensus score ranging from 1 to 4 for imaging quality, with specific attention to motion artifacts. A score of 1 indicated poor quality, 2 indicated sufficient quality, 3 indicated good quality, and 4 indicated excellent quality. Furthermore, the diagnostic validity of each MRI scan was assessed, and the need for an additional MRI scan to address the same clinical question was determined for each subject.

#### 2.1.2 Anesthetic protocol

Patient data were collected from the clinical-anesthesiologic record for each subject. Patient characteristics, including gender, age in days at the time of the scan, clinical indication for MRIwere recorded (refer to Table 3). The safety of the sedation protocol was evaluated based on the assessment of the following parameters collected from the anesthesiologic record:

• Post-anesthesia agitation, defined by a four-point scale based on that of Aono et al. (11), where 1 indicated calmness; 2 indicated being quite calm, 3 indicated agitation, and 4 indicated being very agitated.
• Presence or absence of post-anesthesia nausea and vomiting.
• Time from eye-opening to drinking and eating.
• Time from eye-opening to hospital discharge.
• Changes in vital parameters (heart rate, saturation rate, capnography and blood pressure) during anesthesia and in the post-anesthesia period. Vital parameters were continuously monitored during anesthesia and recorded every 30 min during the post-anesthesia observation period. The recovery time was established based on the Post Anesthetic Discharge Scoring System (12) and it was the same for newborns, infant and children.
• Side effects, such as allergic reactions.
• Need for unplanned hospitalization due to anesthesia.

**Table 3:** Optimized anesthesiologic protocol group characteristics.

	Descriptive statistics
Gender	
- Male	29 (58%)
- Female	21 (42%)
Age (months)	
- Media	40,4
- Range	1–180
- Standard deviation	36,7
Younger than 3 months old but weighed more than 4 kg	11 (12%)
Body part	
- Brain	49 (98%)
- Spine	1 (2%)
Contrast-medium administration	10 (20%)
Indication for scan	
- Follow-up hypoxic-ischemic brain insult - Genetic disorders - Metabolic disorders - Epilepsy - Follow-up brain infection - Trauma - Prematurity - Follow-up in cerebral venous thrombosis - Spinal dysraphism	20 (38,7%) 5 (10.2%) 5 (10.2%) 4 (8,2%) 4 (8,2%) 2 (4,1%) 2 (4,1%) 2 (4,1%) 1 (2%)

#### 2.1.3 Parents’ satisfaction

Our “Good Practice” protocol includes feedback by parents through satisfaction questionnaires. Therefore, the satisfaction questionnaires previously given to parents were collected and analyzed. The questionnaires included three questions:

• For parents of patients undergoing MRI examination with the feed and wrap technique, the evaluation of the opportunity to carry out the MRI examination without anesthesia was rated on a scale from 1 to 4, where 1 indicated unsatisfactory, 2 satisfactory, 3 good, and 4 excellent.
• For parents of patients undergoing MRI examination with the optimized anesthesiologic protocol, the evaluation of the possibility to carry out the MRI examination without child hospitalization was rated on a scale from 1 to 4, where 1 indicated unsatisfactory, 2 satisfactory, 3 good, and 4 excellent.
• The evaluation of the medical and nursing logistics organization pre- and post-MRI execution was rated on a scale from 1 to 4, where 1 indicated insufficient, 2 sufficient, 3 good, and 4 excellent.

### 2.2 Statistical analysis

The characteristics of the patient cohort were analyzed using descriptive statistics, including means and standard deviations (SDs). Frequencies were determined using basic models. Regression analysis was conducted, with a significance level set at a *p*-value of <0.05. The analysis was carried out using SPSS, version 18.

## 3 Results

### 3.1 Feed and wrap technique

A total of 82 feed and wrap scans were identified during the study period. Among the 82 scan sessions conducted, the average patient age was 50 days (range = 1–120 days), with 9.75% being preterm.

The majority of scans (85.36%) were brain MRIs, encompassing axial, coronal, and sagittal T2-weighted images; an axial T1-weighted image; an axial DWI with reconstructed ADC maps; and an axial SWI. Common clinical indications included hypoxic-ischemic brain injury (34.15%), congenital or neonatal infection (14.63%), metabolic disorders (12.19%), and neurological symptoms (24.39%). Other MRI examinations included the spine for suspected dysraphism (7.32%), and the head and neck district (7.32%). Additionally, 2 MR-Spectroscopy (MRS) scans were added to the standard MRI brain protocol. Among all scans, 7 (8.5%) were performed with contrast medium injection.

The neuroradiologists classified the quality of the 82 feed and wrap scans as follows (refer to Table 4): 4 (4.87%) as poor (score 1), 15 (18.29%) as sufficient, 31 (37.80%) as good (score 3), and 32 (39.92%) as excellent (score 4). The injection of contrast did not impact the image quality negatively, with the 7 MRI scans acquired with contrast medium being rated as good or excellent by the readers. According to a univariate logistic regression, a history of preterm birth was associated with a lower MRI quality score (OR = 2.26, *P* = .047 CI95% 1.0083, 5.0679).

**Table 4:** Classification of the quality of the 82 feed and wrap MRI scans.

Quality score	Number of scans (percentage)
1—Poor	4 (4.87%)
2—Sufficent	15 (18.29%)
3—Good	31 (37.8%)
4—Excellent	32 (39.9%)

Two out of the 82 scans required MRI repetition to address the same clinical question, both of which were scored as 1 by neuroradiologists. However, the remaining 2 scans with a score of 1 did not necessitate a supplementary examination because, despite numerous movement artifacts, they still yielded diagnostic result.

No vital parameters alterations were noted before, during, or after the scan.

### 3.2 Anesthetic protocol

In total, 50 patients underwent MRI examination with the optimized anesthetic protocol in the study period. Out of the 50 patients, 29 were boys and 21 were girls, with an average patient age of 40.40 months (range = 1–182 months); 11 (12%) patients were less than 3 months old but weighed more than 4 kg, and 39 (78%) were older than 3 months.

All underwent brain MRI examinations, except for one who had a spine MRI due to clinical suspicion of spinal dysraphism. Common clinical indications for brain MRI included follow-up for hypoxic-ischemic brain insult (38.7%), genetic disorders (18.3%), metabolic disorders (10.2%), follow-up for cerebral infections (8.2%), epilepsy (8.2%), trauma (4.1%), prematurity (4.1%), and follow-up for cerebral venous thrombosis (4.1%).

The median time from eye opening to hospital discharge was 174 min (range: 150–180 min). During this time, children were kept under nursing observation, and vital parameters were monitored.

None of the 50 children included in the study showed adverse effects to sedation, such as allergic reactions, or other major complications, including severe hypotension (systolic blood pressure <60 mmHG and diastolic blood pressure <35 mmHg), prolonged (lasting morethan 60 s) bradycardia, or oxygen desaturation.

None of the children were agitated (grade 3) or very agitated (grade 4) after anesthesia; the majority (86%) were calm (grade 1) during the post-anesthesia period, and 7 children (14%) were classified as quite calm (grade 2).

None of the children experienced post-anesthesia nausea and vomiting, or paradoxical reaction to Midazolam

The time from eye opening to drinking was 60 min, and to eating was 120 min for children weaned from milk. For the 11 infants, the time from eye opening to breast milk or 50% diluted artificial milk was 60 min.

None of the children required hospitalization.

### 3.3 Parents satisfaction

100% of parents whose babies underwent MRI examination with the feed and wrap technique rated the possibility of conducting the MRI examination without anesthesia as excellent (score 4).

85.6% of parents of the 50 patients who underwent MRI examination with the optimized anesthesiologic protocol rated the possibility of conducting the MRI examination without child hospitalization as excellent (score 4), while 13.6% (19 couples of parents) considered this possibility good (score 3). In all these 19 cases, parents had slight doubts about the safety of returning the child home after few hours from anesthesia.

Regarding the medical and nursing logistics organization pre- and post-MRI execution, 47% of parents rated the service as excellent (score 4); 50% rated it as good (score 3), and 3% rated it as sufficient (score 2). The main concerns were about pre- and post-MRI waiting time.

## 4 Discussion

Our retrospective study aimed to evaluate the protocol called “Good Practice” used in our Institution, designed to reduce the need for anesthesia and hospitalization in children undergoing MRI examinations.

The protocol is tailored to each patient, taking into account their age and weight. Specifically, for newborns weighing less than 4 kg or infants younger than 3 months and weighing less than 4 kg, we utilized the “feed and wrap” technique with a vacuum immobilization bag.

The feed and wrap technique proved to be as a valid method for newborn imaging (13–21), offering the possibility to avoid general anesthesia in neonates. Consistent with the literature, our results highlight how the majority of MRI examinations acquired with the feed and wrap method presented an overall high standard of imaging quality (39.9% excellent and 37.8% good). Importantly, even the scans scored as poor or sufficient in terms of quality (about 23% in total) are considered diagnostic, except for two. Indeed, only two of the 82 MRI examinations included in the study were invalidated by motion artifacts to the extent that they required a second MRI examination with anesthesia.

Notably, 8.5% of all scans were performed with contrast medium administration, and in our sample, the injection of contrast medium did not affect image quality. Whereas prematurity was linked to lower MRI quality scores, we can speculate that it could be correlated with the difficulty of firmly wrapping small premature newborns, who are thus easily able to move in the vacuum bag.

The positive outcomes observed in the majority of feed and wrap scans underscore the clinical utility of this technique in neonatal neuroimaging. It offers significant advantages by avoiding the need for sedation in these patient groups, thereby preventing hospitalization or other uncomfortable conditions such as pre-examination fasting.

The optimized anesthesiologic protocol confirmed the literature data on propofol's favorable outcomes in terms of safety and efficient recovery. A recent meta-analysis, which included 30 studies comprising 3,774 children who received propofol, highlighted how propofol sedation had advantages in recovery time compared to other anesthetics, without excessive concerns for cardiovascular or respiratory adverse events. The authors concluded that the overall evidence suggests that propofol could be considered as an option for sedation in pediatric procedures (8). Moreover, one of the principal aspects studied in the literature is the low rate of post-anesthesia agitation using Propofol compared to other drugs, such as the widely used Sevoflurane (5, 6), and our results confirmed these data, with significant implications for children's comfort and hospital discharge. Finally, the absence of adverse effects and complications, coupled with the quick recovery of normal activities, allows for the avoidance of hospitalization in these patients.

We believe that integrating parents’ feedback is crucial in evaluating the overall success of our proposed protocol. Regarding the possibility of avoiding anesthesia through the feed and wrap method, the unanimous positive response from parents underscores the efficacy and acceptability of this technique not only for the young patients but also for their parents. Concerning the option of conducting the MRI examination without child hospitalization if anesthesia is required, the majority of parents accepted this option positively. However, a small percentage of parents expressed slight doubts about the safety of returning the child home soon after anesthesia, highlighting a specific concern that warrants attention in the explanation of anesthetic protocols and their potential side effects.

Regarding the medical and nursing logistics organization before and after MRI execution, the overall organization is generally well-received. However, it has emerged that there may be slight discomfort for parents and children, as they are required to stay in the MRI recovery rooms for a few hours after sedation or before undergoing the feed and wrap technique. This slight discomfort could be alleviated by making the recovery rooms as comfortable as possible. Furthermore, avoiding hospitalization for these children may have a positive impact on health costs and hospital organization, allowing more space for children who require hospitalization for other reasons.

Our study has several limitations: first, the study did not include a control group, making it purely an observational and descriptive evaluation of our Hospital protocol, second it is a retrospective study therefore some slight side effect of anesthesia may be not reported in the clinical record and missed in our analysis;. Further research and larger-scale studies may help to validate and refine this protocol. Moreover, the excellent results obtained in our Institute may be attributed to the highly specialized nursing and medical team employed in this protocol. However, the numerous benefits arising from our protocol suggest that it could serve as a starting point for other less specialized facilities that need to perform MRI examinations in the pediatric population.

In conclusion, after analyzing the “Good Practice” protocol used in our Institute, we found that the feed and wrap technique is a valid method for obtaining high-quality MRI scans while avoiding anesthesia, with a low failure rate. Additionally, anesthesia with propofol in the pediatric population was not associated with side effects in our sample, allowing us to achieve the goal of avoiding hospitalization, which was a relief for parents.

## Data Availability

The raw data supporting the conclusions of this article will be made available by the authors, without undue reservation.
